# Multi-Party Quantum Private Comparison Based on Bell States

**DOI:** 10.3390/e25081156

**Published:** 2023-08-02

**Authors:** Wanqing Wu, Jiahui Wu, Lingna Guo

**Affiliations:** 1 School of Cyber Security and Computer, Hebei University, Baoding 071002, China; 2 Key Laboratory on High Trusted Information System in Hebei Province, Hebei University, Baoding 071002, China

**Keywords:** multi-party quantum private comparison (MQPC), Bell state, unitary operation

## Abstract

Multi-party quantum private comparison (MQPC) assumes responsibility for overseeing the flow of data and communication among diverse entities, wherein it boasts powerful security capabilities that have garnered substantial attention. Most current MQPC protocols rely on difficult-to-prepare quantum states and are inefficient in their use of resources. In this paper, we propose a novel MQPC protocol without entanglement swapping, thereby building upon the assumption of an ideal channel. This protocol is based on Bell states, which simplifies implementation and addresses the challenges associated with using complex quantum states; it also enables the comparison of secret information by having a trusted party prepare and transmit encoded quantum sequences to participants, thereby facilitating efficient equality comparison among all parties. Our MQPC protocol showcased remarkable efficiency in comparison to existing protocols for quantum private comparison. Furthermore, the incorporation of decoy photon and shared key technologies made external and internal attacks ineffective, thereby ensuring the utmost security and integrity of the protocol.

## 1. Introduction

Secure multi-party computing (SMC) holds significant prominence within the realm of classical cryptography, with its origins in the millionaire problem that was first ever introduced by Yao [1] in 1982. This fascinating problem revolves around securely comparing the wealth of two wealthy individuals without revealing their actual fortunes. SMC assumes a pivotal role in preserving secrecy in diverse domains, thus encompassing clandestine bidding and auctions, confidential ballot elections, e-commerce, and private data analysis.

Nevertheless, the advent of quantum information processing technology in recent years has introduced vulnerabilities to classical SMC, which relies on complex mathematical puzzles. As a result, efforts have been made to extend SMC to the quantum realm, thereby aiming to address the security limitations inherent in classical SMC. In 1984, Bennett and Brassard [2] amalgamated the principles of quantum mechanics with classical cryptography, thus presenting the pioneering quantum cryptography protocol. Subsequently, a multitude of quantum cryptography protocols have been put forward, which encompass quantum secret sharing (QSS) [3,4], quantum key distribution (QKD) [2,5,6,7,8,9,10], quantum private query (QPQ) [11,12], quantum-secure direct communication (QSDC) [13,14], and various others.

QKD is a quantum communication protocol that is used for key distribution, where the quantum states of qubits are employed to transmit the keys. It can detect any eavesdropping or interference in the key transmission to ensure the security of the keys. To detect the presence of eavesdroppers, QKD introduces the concept of decoy states [15,16,17]. This is a special quantum bit state that is injected into the communication channel during transmission and is mixed with the transmitted key bits. If an eavesdropper attempts to interfere with the communication and measures the transmitted quantum bits, they inevitably affect the state of the decoy state. Therefore, decoy photon technology is often used to ensure the security of key distribution.

By employing QKD for secure quantum key distribution, quantum secure multi-party computing (QSMC) enables confidential communication among multiple users while providing efficient multi-plexing capabilities.

The quantum privacy comparison protocol (QPC), which is a prominent component of the QSMC protocol, has garnered significant interest. Its primary goal is to determine the equality of confidential values among participants while preserving the secrecy of the information. The groundbreaking QPC protocol introduced by Yang and Wen [18] has greatly facilitated various QPC protocols that utilize different quantum states as resources, including protocols based on single photons [19], Bell states [20,21,22], GHZ states [23,24], and multi-particle entangled states [25,26,27], among others. However, it is always necessary to perform the protocol several times when comparing the secret information of multiple participants when using two-party QPC protocols. To address this limitation, Chang et al. [28] proposed the original Multi-Party Quantum Private Comparison Protocol (MQPC) in 2013, thereby enabling the comparison of several secret information in a single execution. This protocol employed an *n*-particle GHZ state and facilitated the comparison of secret information among *n* participants. Since then, significant research efforts have been dedicated to advancing the MQPC.

In 2016, Ye [29] introduced an MQPC protocol that utilized the entanglement swapping of Bell-entangled states. The following year, Ji and Ye [30] proposed another MQPC protocol that employed the entanglement swapping of *d*-level cat states and *d*-level Bell states. Later in 2019, Ye [31] presented an MQPC protocol that utilizes single-particle states with multiple levels. It is evident from this analysis that most existing MQPC protocols rely on entangled states of *n* particles or *d*-level systems. However, the preparation and measurement of *d*-level systems and *n*-particle-entangled states remain challenging within current quantum technologies. These proposed solutions are currently only theoretically feasible and require significant advancements in quantum technology for their practical implementation. Another issue arises from the inefficiency of the existing MQPC protocols, thus resulting in sub-optimal utilization of the quantum resources and significant wastage.

To address the aforementioned concerns, we have developed an MQPC protocol that utilizes the Bell state as the foundational quantum resource. This protocol effectively simplifies the implementation process by tackling the challenge of using complex quantum states to achieve the comparisons across n participants. Additionally, it demonstrated superior quantum efficiency when compared to most existing MQPC protocols.

The remaining portion of this manuscript is well-structured, with the proposed protocol as the central focus. In Section 2, the fundamental concepts involved are introduced. Section 3 provides a detailed description of the protocol, including an analysis of its accuracy, and, in Section 4, the security of it is analyzed. In Section 5, a comparison is made between the proposed protocol and several other protocols that utilize the TP. Ultimately, Section 6 provides a summary and concludes the paper.

## 2. Background

The Bell states, as denoted in Equation (1):

(1) $$|\phi^{\pm }\rangle =\frac{1}{\sqrt{2}}(|00\rangle \pm \left|11\rangle ),\right|\psi^{\pm }\rangle =\frac{1}{\sqrt{2}}(|01\rangle \pm |10\rangle ).$$

The given values constitute a pair of entangled states involving two particles exhibiting maximal entanglement. Furthermore, they serve as a comprehensive and mutually perpendicular basis within a four-dimensional Hilbert space. In this paper, the Bell state was adopted as a quantum carrier.

The entanglement of the two particles in the Bell state signifies that the application of a unitary operation on an individual particle within the Bell state will invariably bring about a modification in the other particle. We have four unitary operations available—*X*, *Y*, *Z*, and *I*—which are defined as follows:

(2) $$\begin{array}{c} Z=|0\rangle \langle 0|-|1\rangle \langle 1|,\;X=|0\rangle \langle 1|+|1\rangle \langle 0|,\\ Y=|0\rangle \langle 1|-|1\rangle \langle 0|,\;I=|0\rangle \langle 0|+|1\rangle \langle 1|. \end{array}$$

When any one of the operations *I*, *X*, *Y*, or *Z* is applied to the first particle of a given Bell state, it induces a transformation that manifests in two Bell states. The inter-relationship governing this transformation is elucidated in Table 1.

Using the above Equation (2), the iterative results of the unitary operations X,Y,Z, and *I* are shown in Table 2.

## 3. The Proposed Scheme

### 3.1. Prerequisites

The proposed protocol was conducted under the ideal condition of noiselessness. Prior to presenting this protocol, it is necessary to outline its prerequisites in the following:

1. Let $\left\{P_{i}\;|\;i=1,2,\cdots ,n\right\}$ represent *n* parties holding different private information $M_{i}=m_{i}^{1}m_{i}^{2}\cdots m_{i}^{L}$, respectively, where $m_{i}^{j}\in \left\{0,1\right\},j=1,2,\cdots ,L$. Being aided by a semi-honest TP, their primary aim is to determine if their respective secrets bear resemblance. The TP is allowed to exert effort in recognizing the content of the participants’ secrets, but it is prohibited from colluding with others.
2. $M_{i}$ is divided into sets $\left\{G_{i}^{1},G_{i}^{2},\cdots ,G_{i}^{\left\lceil \frac{L}{2}\right\rceil }\right\},$ in which each $G_{i}^{j}$ consists of two bits: $j=1,2,\cdots ,\lceil \frac{L}{2}\rceil$. In the case where *L* mod 2≠0, the last set $G_{i}^{\left\lceil \frac{L}{2}\right\rceil }$ is padded with 0 for completeness.
3. Through the implementation of a multi-party QKD [32], two types of key sequences, denoted as $K_{P}$ and $K_{TP_{i}}$, are generated. In particular, $K_{P}$ serves as the secret key shared among all participants $P_{i}$, while $K_{TP_{i}}$ signifies the key that is mutually shared between the TP and $P_{i}$:
  $$\begin{array}{ccc} K_{P} & =\left(k_{P}^{1},k_{P}^{2},\cdots ,k_{P}^{\left\lceil \frac{L}{2}\right\rceil }\right),\;K_{TP_{i}} & =\left(k_{TP_{i}}^{1},k_{TP_{i}}^{2},\cdots ,k_{TP_{i}}^{\left\lceil \frac{L}{2}\right\rceil }\right). \end{array}$$
  where, $k_{p}^{j},k_{TP_{i}}^{j}\in \left\{00,01,10,11\right\}$, $j=1,2,\cdots ,\lceil \frac{L}{2}\rceil$.
4. In this paper, the quantum carrier is taken as Bell state
  $$|\phi^{\pm }\rangle =\frac{1}{\sqrt{2}}(|00\rangle \pm \left|11\rangle ,\;\right|\psi^{\pm }\rangle =\frac{1}{\sqrt{2}}(|01\rangle \pm |10\rangle .$$

### 3.2. Protocol Steps

The proposed MQPC protocol will be comprehensively explained in this section.

**Step 1**

The TP prepares a total of $n\cdot \lceil \frac{L}{2}\rceil$ quantum states by selecting from the set $\{|\phi^{\pm }\rangle ,|\psi^{\pm }\rangle \}$. These states are used to construct *n* sequences of quantum states denoted that are as $\left\{S_{i}\;|\;i=1,2,\cdots ,n\right\}$. $S_{i}$ can be expressed as follows:

$$S_{i}=\left(Q_{P_{i}}^{1}Q_{TP_{i}}^{1},Q_{P_{i}}^{2}Q_{TP_{i}}^{2},\cdots ,Q_{P_{i}}^{\left\lceil \frac{L}{2}\right\rceil }Q_{TP_{i}}^{\left\lceil \frac{L}{2}\right\rceil }\right),$$

in which the superscripts $1,2,\cdots ,\lceil \frac{L}{2}\rceil$ indicate the position of the Bell state in the $S_{i}$. Furthermore, the indices $P_{i},TP_{i}$ distinguish the different particles within each Bell state.

Subsequently, two new sequences are formed by extracting out the first and second particles of each Bell state, respectively:

$$\begin{array}{ccc} S_{P_{i}} & =\left(Q_{P_{i}}^{1},Q_{P_{i}}^{2},\cdots ,Q_{P_{i}}^{\left\lceil \frac{L}{2}\right\rceil }\right),\;S_{TP_{i}} & =(Q_{TP_{i}}^{1},Q_{TP_{i}}^{2},\cdots ,Q_{TP_{i}}^{\left\lceil \frac{L}{2}\right\rceil }). \end{array}$$

For each i∈[1,n], one advances in a sequential manner by executing Step 2, Step 3, and Step 4 successively.

**Step 2**

The TP generates *n* decoy sets, each consisting of elements from a basis set {|0〉,|1〉,|+〉,|−〉}, where:

(3) $$|\pm \rangle =\frac{1}{\sqrt{2}}(|0\rangle \pm |1\rangle ).$$

These decoy sets are then inserted randomly by the TP into the sequence $S_{P_{i}}$, thereby resulting in a new sequence that is identified as the $S_{P_{i}}^{*}$. The TP subsequently transmits the modified sequence $S_{P_{i}}^{*}$ to the participant $P_{i}$ in order to effectively detect any potential eavesdropping.

Once it is verified that the $P_{i}$ has received all the particles of the $S_{P_{i}}^{*}$, the TP continues to verify that the transmissions from the $S_{P_{i}}^{*}$ to the $P_{i}$ were secure through several steps. Firstly, the TP informs the $P_{i}$ about the location and preparation basis of the decoy photons in the $S_{P_{i}}^{*}$. The $P_{i}$ then measures the decoy photons and feeds back the results to the TP. Finally, the TP compares the measurements with the initial state to calculate the error ratio. If the error ratio is above a predefined threshold level, the communication will be halted, and the protocol procedure will be reset. Otherwise, the $P_{i}$ will discard the decoy photons in the $S_{P_{i}}^{*}$ to resume the $S_{P_{i}}$, and the protocol advances to the next step.

**Step 3**

The $P_{i}$ utilizes the shared keys $k_{P}^{j}$ and $k_{TP_{i}}^{j}$ to encrypt the confidential information $G_{i}^{j}$ through an exclusive OR operation, thereby yielding $R_{i}^{j}=G_{i}^{j}⊕k_{P}^{j}⊕k_{TP_{i}}^{j}$.

In adherence to the coding regulations outlined in Table 3, the $P_{i}$ chooses a unitary operation based on the value of the $R_{i}^{j}$, which is denoted as $U_{R_{i}^{j}}$, which is applied on the *j*th particles of the $S_{P_{i}}$. This modification affects the states of the particles in the $S_{P_{i}}$, thereby resulting in a transformed sequence that is labeled as $S_{P_{i}}^{\prime }$.

Subsequently, the $P_{i}$ inserts a collection of decoy photons, each belonging to the basis sets {|0〉,|1〉,|+〉,|−〉}, into various positions of the transformed sequence $S_{P_{i}}^{\prime }$. This construction forms a novel sequence that is identified as $S_{P_{i}}^{\prime *}$.

Finally, the $P_{i}$ transmits the sequence $S_{P_{i}}^{\prime *}$ to $P_{i+1}$, with particular emphasis on the fact that $S_{P_{n}}^{\prime *}$ will be sending to $P_{1}$.

**Step 4**

Upon confirming $P_{i+1}$’s reception of all the particles, the $P_{i}$ proceeds to verify the security of transmitting $S_{P_{i}}^{\prime *}$ to $P_{i+1}$ through a procedure that is similar to Step 2. If the ratio surpasses the pre-determined threshold level, the communication will be terminated, and the protocol procedure will be restarted. Otherwise, the communication will continue.

The $P_{i+1}$ then removes the decoy photons in the $S_{P_{i}}^{\prime *}$ to restore the original form of the $S_{P_{i}}^{\prime }$. Subsequently, the unitary operation $U_{R_{i+1}^{j}}$, which is determined by $R_{i+1}^{j}$ and the mapping rules in Table 3, is applied to the *j*th particles of the $S_{P_{i}}^{\prime }$, thereby transforming $S_{P_{i}}^{\prime }$ into $S_{P_{i}}^{\prime \prime }$.

For utmost security, the $P_{i+1}$ prepares a set of decoy photons consisting of elements from the basis sets {|0〉,|1〉,|+〉,|−〉} randomly. These decoy photons are then inserted randomly into the $S_{P_{i}}^{\prime \prime }$, thereby creating a new sequence called $S_{P_{i}}^{\prime \prime *}$. Finally, the $P_{i+1}$ transmits the $S_{P_{i}}^{\prime \prime *}$ to the TP.

**Step 5**

Upon authenticating the TP’s reception of all the particles, the $P_{i+1}$ verifies the security of transmitting the $S_{P_{i}}^{\prime \prime *}$ to the TP, thus following a similar procedure as the describe in Step 2. If the ratio surpasses a pre-defined threshold, the transmission will be halted, and the protocol procedure will be reset. Otherwise, the communication will persist uninterrupted.

The TP discards the decoy photons within the $S_{P_{i}}^{\prime \prime *}$ and restores the sequence as $S_{P_{i}}^{\prime \prime }$. Next, it selects the unitary operation $U_{k_{TP_{i}}^{j}⊕k_{TP_{i+1}}^{j}}$ that is determined by the value of $k_{TP_{i}}^{j}⊕k_{TP_{i+1}}^{j}$ and the rules presented in Table 3, and applies it to the *j*th particles of the $S_{P_{i}}^{\prime \prime }$. Consequently, the sequence $S_{P_{i}}^{\prime \prime }$ undergoes transformation into $S_{P_{i}}^{\prime \prime \prime }$.

Subsequently, the particles in both the $S_{P_{i}}^{\prime \prime \prime }$ and $S_{TP_{i}}$ sequences are subjected to Bell basis measurement. By comparing the measurement results of each Bell state to the initially prepared states, the TP can verify if any changes have occurred: if the measurements align perfectly with the primary states, the TP will declare that $M_{1},M_{2},\cdots ,M_{n}$ are all equal; if those results differ from the initial states, the TP will announce that $M_{1},M_{2},\cdots ,M_{n}$ are not all equal.

**Figure 1:**
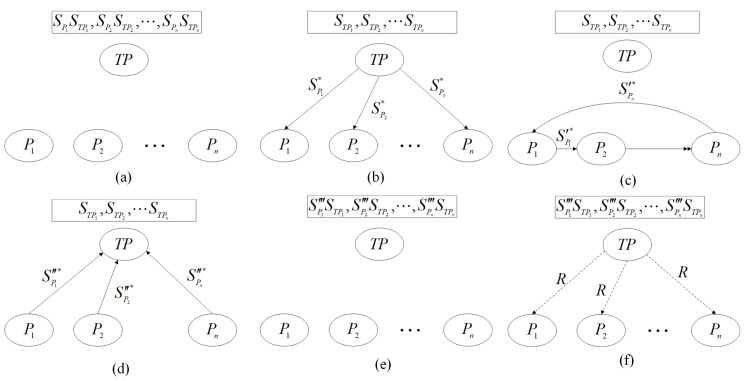
The proposed protocol process is depicted in a simple flow chart, where quantum channels are represented by solid arrow lines, classical channels by dashed arrow lines, and multiple transmissions by double arrows. To maintain clarity, we exclude any considerations regarding external eavesdropping and eavesdropping inspection steps. (**a**) TP-prepared *n* sets of quantum states sequences $\left\{S_{i}\;|\;i=1,2,\cdots ,n\right\}$ and each $S_{i}$ separated into two sequences $S_{P_{i}}$ and $S_{TP_{i}}$. (**b**) TP transmits the sequence $S_{P_{i}}^{*}$ to $P_{i}$ while retaining the sequence $S_{TP_{i}}$. (**c**) $P_{i}$ delivers the unitary operation sequences $S_{P_{i}}^{\prime *}$ to $P_{i+1}$. (**d**) $P_{i+1}$ delivers the unitary operation sequences $S_{P_{i}}^{\prime \prime *}$ to TP. (**e**) The TP performs a unitary operation on the sequence $S_{P_{i}}^{\prime \prime }$, thus resulting in a modified sequence denoted as $S_{P_{i}}^{\prime \prime \prime }$. (**f**) Bell basis measurement is employed to measure the sequences $S_{TP_{i}}$ and $S_{P_{i}}^{\prime \prime \prime }$, followed by the comparison of the obtained measurements against the $P_{i}$ to determine the comparative result *R*. *R* is then declared.

For the sake of clarity, a flowchart representing the entire protocol can be found in Figure 1.

### 3.3. Correctness

In the aforementioned steps, all the $P_{i}$ examine the equality of their private information $M_{i}$ with the cooperation of the TP. During Step 5, the TP scrutinizes the measurements in relation to the original records to determine if the secrets are indeed identical. The confirmation of their identity takes place when the measurements align, whereas any disparities in the measurements indicate non-identity.

We present the validity of the proposed protocol through the following details. Let us consider the *j*th particle $Q_{P_{i}}^{j}Q_{TP_{i}}^{j}\left(j=1,\cdots ,\left\lceil \frac{L}{2}\right\rceil \right)$ in $S_{i}$. After all the parties of the $P_{i}$ complete the unitary operations of the presented protocol on the initial particle $Q_{P_{i}}^{j}Q_{TP_{i}}^{j}$, its state is transformed to the following form:

(4) $$U_{k_{TP_{i}}^{j}⊕k_{TP_{i+1}}^{j}}U_{R_{i+1}^{j}}U_{R_{i}^{j}}⊗I\left(Q_{P_{i}}^{j}Q_{TP_{i}}^{j}\right)=U_{G_{i}^{j}⊕G_{i+1}^{j}}⊗I\left(Q_{P_{i}}^{j}Q_{TP_{i}}^{j}\right).$$

Upon referring to Table 2 and Table 3, we observe that the unitary operations $U_{R_{i}^{j}}$ and $U_{R_{i+1}^{j}}$ adhere to the commutative law:

(5) $$U_{R_{i+1}^{j}}U_{R_{i}^{j}}=U_{R_{i+1}^{j}⊕R_{i}^{j}}=U_{R_{i}^{j}⊕R_{i+1}^{j}}=U_{R_{i}^{j}}U_{R_{i+1}^{j}}.$$

By substituting $R_{i}^{j}=G_{i}^{j}⊕k_{P}^{j}⊕k_{TP_{i}}^{j}$ and $R_{i+1}^{j}=G_{i+1}^{j}⊕k_{P}^{j}⊕k_{TP_{i+1}}^{j}$ into Equation (5), we conclude the following:

(6) $$U_{R_{i+1}^{j}}U_{R_{i}^{j}}=U_{R_{i}^{j}}U_{R_{i+1}^{j}}=U_{G_{i}^{j}⊕k_{P}^{j}⊕k_{TP_{i}}^{j}⊕G_{i+1}^{j}⊕k_{P}^{j}⊕k_{TP_{i+1}}^{j}}=U_{G_{i}^{j}⊕k_{TP_{i}}^{j}⊕G_{i+1}^{j}⊕k_{TP_{i+1}}^{j}}.$$

In Step 5, the TP performs the unitary operation $U_{k_{TP_{i}}^{j}⊕k_{TP_{i+1}}^{j}}$ on the *j*th particle. According to Equation (6),

$$U_{k_{TP_{i}}^{j}⊕k_{TP_{i+1}}^{j}}U_{R_{i+1}^{j}}U_{R_{i}^{j}}=U_{k_{TP_{i}}^{j}⊕k_{TP_{i+1}}^{j}⊕G_{i}^{j}⊕k_{P}^{j}⊕k_{TP_{i}}^{j}⊕G_{i+1}^{j}⊕k_{P}^{j}⊕k_{TP_{i+1}}^{j}}=U_{G_{i}^{j}⊕G_{i+1}^{j}}$$

is derived, thereby confirming the validity of Equation (4). It allows the TP to determine the participants’ secret equality by comparing measurements with the initial state. If $G_{i}^{j}=G_{i+1}^{j}$, the initial state matches the measured state, thereby indicating correctness, while a disparity signifies a difference. Thus, the presented protocol functions correctly in enabling the TP to easily ascertain the equality of all the participants’ secrets.

## 4. Security Analysis

We undertake an analysis of the security aspects pertaining to the proposed protocol in this section. Firstly, we state that any external attacks directed at the protocol are rendered ineffectual. Secondly, it becomes evident that participants, whether individually or in a group of n−1, are unaware of the confidential information held by other parties. Finally, the TP has the authority to disclose comparison outcomes, but it cannot access to any confidential information from other participants. As a result, this protocol guarantees privacy while facilitating the comparison of the secret equality among participants.

### 4.1. Outsider Attack

The potential of an external eavesdropper named Eve to gain the confidential information of *n* parties is the central focus of this comprehensive analysis. It is worth noting that the assumed security in our QKD technique effectively prevents Eve from accessing the shared keys.

Our protocol effectively eliminates Eve’s opportunity to launch an attack in Step 1, which does not involve any qubit transmissions. However, Steps 2, 3, and 4 comprise qubit transmission over the quantum channel that Eve could take advantage of to gather the secrets of *n* parties. Such attacks might involve the intercept-resend attack, the measure-resend attack, the entangle-measure attack, or related standard methods. Nevertheless, our proposed protocol incorporates the use of decoy photon technology, thereby guaranteeing the security of the quantum transmission. This technique is a variation of the eavesdropping detection method employed in the BB84 quantum key distribution protocol [2], which has been mathematically proven to have unconditional security [33].

In Step 5, our protocol effectively neutralizes Eve’s ability to launch any attacks while processing the information locally. As a result, the external eavesdropper can only acquire knowledge of the comparison result, thereby making her pursuit to acquire the secrets of the $P_{i}$ entirely futile.

While this protocol is formulated based on the ideal scenario, noise remains an inevitable factor in quantum systems. In practical quantum noise channels, the noise present in the quantum channels may potentially affect the particles, thus leading to quantum bit errors. Error suppression techniques, such as dynamic decoherence suppression, can effectively alleviate the impact of noise on the quantum bits, thereby reducing the overall transmission error rate. Additionally, quantum error-correcting codes are beneficial for detecting and correcting errors in the quantum bits during transmission. Hence, by integrating error suppression techniques and quantum error-correcting codes, we can ensure the robustness and security of this protocol in noisy environments.

To conclude, we confidently assert that the presented protocol has undergone a thorough analysis and incorporates measures at each step to mitigate vulnerabilities, thereby ensuring its resilience to external attacks.

### 4.2. Insider Attack

In their 2007 study, Gao et al. [34] proposed that attacks originating from participants who are actively engaged in implementing a protocol could pose a substantial threat regarding security. This raises the crucial question of whether the protocol under scrutiny possesses the ability to withstand internal attacks.

***Case 1: Individual Attacks of Participants***

Considering the possibility of a malicious participant, denoted as $P_{1}$, who aims to access others’ secrets, it is imperative to contemplate any potential attacks that may transpire during the transmission of the particles across the quantum channel. Nevertheless, any efforts by the $P_{1}$ to intercept these particles will undoubtedly trigger detection by the eavesdropping check processes, as such attacks are bound to leave discernible traces on the decoy photons.

Another avenue through which the $P_{1}$ could attempt to obtain confidential information is by analyzing the sequence of the quantum states within his or her possession. Although the $P_{1}$ may possess knowledge of the $S_{P_{n-1}}^{\prime }$ during Step 4, this knowledge alone is inadequate for extracting the $P_{n-1}$’s secret. This limitation arises from the fact that the $P_{1}$ lacks access to the shared key $K_{TP_{n-1}}$ or the initial Bell state that is prepared by the TP.

Therefore, the evidence suggests that dishonest participants, such as the $P_{i}$, are effectively prevented from acquiring the secrets of others.

***Case 2: Conspiracy Attack Among Participants***

Our primary focus is directed toward the most extreme scenario, where a group of dishonest parties conspires to obtain the secret held by a single user, thus excluding all other participants. This specific case represents the greatest threat to the overall agreement.

To provide clarity, let us consider a hypothetical situation where the dishonest parties, which are referred to as $P_{1},P_{2},\cdots ,P_{i-1},P_{i+1},\cdots ,P_{n}$, collaborate with the intention of stealing the secret belonging to the user $P_{i}$.

In order to access the secret information, these parties would need to obtain two separate values: $K_{P}$ and $K_{TP_{i}}$. While the $K_{P}$ serves as a shared key among all participants, dishonest parties may be aware of its existence. However, their knowledge is restricted to the $K_{P}$ alone, as they do not have access to the specific key $K_{TP_{i}}$ that is exclusively shared between the user $P_{i}$ and the TP; thus a trusted third party is renowned for its impartiality and non-collusion with other participants.

Consequently, we can confidently assert that the malevolent parties, comprising $P_{1},P_{2},\cdots ,P_{i-1},P_{i+1},\cdots ,P_{n}$, are rendered futile in their attempt to pilfer the secret belonging to the user $P_{i}$.

***Case 3: Attacks From the TP***

The initial requirement of our protocol mandates the TP to act as a semi-honest third party. The TP’s role is to make every effort to gain access to the participants’ confidential data while ensuring that they do not engage in any collusion with either party.

Suppose a suppositional scenario where the TP covertly attempts to obtain a $P_{i}$’s secret with hidden motives and surprisingly manages to acquire the $S_{P_{i}}^{\prime \prime }$ from the $P_{i+1}$ undetected. Nevertheless, despite having access to the $R_{i}^{j}⊕R_{i+1}^{j}$ obtained by measuring the $S_{P_{i}}^{\prime \prime }$, the TP lacks the necessary knowledge to deduce the true secret belonging to the $P_{i}$ due to the encryption method utilized for the $G_{i}$, which is encrypted by both the $K_{P}$ and $K_{TP_{i}}$. Moreover, any bold attempt by the TP to intercept the particles transmitted via the quantum channel would promptly trigger a vigilant array of detection mechanisms, thereby definitively revealing the TP as an external attacker.

Consequently, the proposed protocol successfully foils the TP’s malicious intentions, thus ensuring the unwavering integrity and security of the system.

## 5. Comparison

Currently, a few protocols exist that intend to bolster security and broaden the functionalities of multi-party quantum computation by diminishing or eliminating reliance on the trusted TP. Nevertheless, in most MQPC protocols, the TP is still employed, especially when a dependable quantum-secure communication channel is unavailable or unworkable. Consequently, our comparison is constrained to protocols that incorporate the involvement of the TP in the latter scenario.

A comparative analysis was conducted on various existing MQPC protocols that employ quantum carriers such as Bell states and other quantum states. The comprehensive findings of this analysis are available in Table 4 and Table 5. In this evaluation, the efficiency of the qubits is denoted as $\eta_{e}$ and shown in Equation (7):

(7) $$\eta_{e}=\frac{\eta_{c}}{\eta_{q}},$$

where $\eta_{c}$ represents the classical bits consumed in comparison, and $\eta_{q}$ denotes the total particles consumed during each comparison phase. Notably, our assessment did not consider the decoy photons and keys produced by QKD.

The proposed protocol demonstrates several advantages compared to the existing MQPC protocols. By incorporating the protocols listed in Table 4, our approach utilizes the Bell state as quantum carriers and presents significant enhancements over the current MQPC protocols. One notable advantage is its superior quantum efficiency, which becomes evident upon examining Table 4. Our protocol achieves twice the qubit efficiency of Refs. [35,36], and achieves three times the efficiency of Ref. [30]. Moreover, our protocol surpasses the quantum resources and measurement capabilities of Refs. [30,36]. This superiority stems from the simplicity with respect to preparing and measuring two-level Bell states, as opposed to the more intricate procedures involved in preparing and measuring *d*-level Bell-entangled states. Additionally, it is worth noting that the comparison between the MQPC protocols relies on different quantum technologies. While both Refs. [30,36] utilized quantum entanglement swapping and unitary operations, our protocol exclusively relies on unitary operations.

We also compared our presented protocol with protocols involving multi-particle entangled states [37,38] and *d*-level systems [31,39]. The comparison results are shown in Table 5. It can be inferred that using Bell states as quantum resources to some extent makes the MQPC protocol feasible. These quantum states can be generated without expensive equipment and complex methods, thereby allowing for a high efficiency and low cost. On the other hand, the preparation and measurement of *n*-particle-entangled states and *d*-level systems may still be challenging with current quantum technologies. Fortunately, our proposed protocol only requires Bell-entangled states and unitary operations on single particles, and these techniques have been successfully demonstrated in experiments [40,41]. In contrast to Refs. [30,31,36,37,38,39], our protocol is based on two-dimensional photons instead of *d*-dimensional photons and *n*-particle-entangled states. This fundamental difference makes our protocol more feasible and practical.

## 6. Conclusions

In conclusion, based on the noiseless conditions, we have devised an innovative MQPC protocol that leverages Bell states, thereby eliminating the need for entanglement swapping. This groundbreaking protocol allows for the secure comparison of secrets among multiple participants within a single execution. By using decoy photons to prevent eavesdropping, encrypting the secrets of participants with shared keys, and employing a sequence of four unitary operations to encode the information into quantum states, our protocol guarantees robust security.

Extensive analysis and scrutiny have unequivocally validated the impervious nature of this protocol against both external and internal attacks. Moreover, our protocol presents noteworthy advantages over its predecessors in the realm of MQPC protocols, particularly concerning the quantum state preparation and overall efficiency. Another salient aspect worth mentioning is that the proposed protocol can be readily implemented by employing existing quantum technologies.

## Figures and Tables

**Table 1:** Transformation relation.

	$|\phi^{+}\rangle$	$|\phi^{-}\rangle$	$|\psi^{+}\rangle$	$|\psi^{-}\rangle$
*X*	$|\psi^{+}\rangle$	−$|\psi^{-}\rangle$	$|\phi^{+}\rangle$	−$|\phi^{-}\rangle$
*Y*	$|\psi^{-}\rangle$	−$|\psi^{+}\rangle$	$|\phi^{-}\rangle$	$|\phi^{+}\rangle$
*Z*	$|\phi^{-}\rangle$	$|\phi^{+}\rangle$	$|\psi^{-}\rangle$	$|\psi^{+}\rangle$
*I*	$|\phi^{+}\rangle$	$|\phi^{-}\rangle$	$|\psi^{+}\rangle$	$|\psi^{-}\rangle$

**Table 2:** Unitary operation rule.

	*I*	*Z*	*X*	*Y*
*I*	*I*	*Z*	*X*	*Y*
*Z*	*Z*	*I*	*Y*	*X*
*X*	*X*	−Y	*I*	−Z
*Y*	*Y*	−X	*Z*	*I*

**Table 3:** The coding rule.

$R_{i}^{j}$	$U_{R_{i}^{j}}$
00	*I*
01	*Z*
10	*X*
11	*Y*

**Table 4:** Comparison with the other protocols based on Bell states.

	Ref. [35]	Ref. [29]	Ref. [36]	Ref. [30]	Our Protocol
Quantum state	Bell state	Bell state	*d*-particle Bell state	n+1-particle cat state and two-particle Bell state both in *d*-level	Bell state
The quantum measurement base of Tp	Single particle	Bell basis	*d*-level two-particle Bell basis	*d*-level n+1-particle cat state	Bell basis
The quantum measurement base of parties	Single particle	Bell basis	*d*-level two-particle Bell basis	*d*-level two-particle Bell basis	×
QKD	*√*	×	×	×	*√*
Unitary operation	×	×	*√*	*√*	*√*
Entanglement swapping	×	*√*	*√*	*√*	×
Qubit efficiency	$\frac{1}{2n}$	$\frac{1}{n+1}$	$\frac{1}{2n+2}$	$\frac{1}{3n+1}$	$\frac{1}{n}$

**Table 5:** The comparison of the presented protocol with respect to the other protocols.

	Ref. [31]	Ref. [37]	Ref. [38]	Ref. [39]	Our Protocol
Quantum state	*n*-level single-particle state	*n*-particle GHZ state	Three-qubit-entangled states	*n*-level single-particle state	Bell state
The quantum measurement base of Tp	*n*-level single-particle state	×	Single particle	*n*-level single particle	Bell basis
The quantum measurement base of parties	×	Single particle	Single particle	×	×
QKD	*√*	×	*√*	*√*	*√*
Quantum technology	Unitary operation	×	×	QFT and phase operation	Unitary operation

## Data Availability

The manuscript has no associated data.
